# Iodine Intake and Testosterone

**DOI:** 10.1001/jamanetworkopen.2023.48573

**Published:** 2023-12-20

**Authors:** Arcangelo Barbonetti, Chiara Castellini, Francesca Di Giulio, Federica Antolini, Daniele Tienforti, Mario Muselli, Marco Giorgio Baroni

**Affiliations:** 1 Andrology Unit, Department of Clinical Medicine, Life, Health and Environmental Sciences, University of L’Aquila, L’Aquila, Italy; 2 IRCCS Neuromed, Pozzilli (IS), Italy; 3 Department of Life, Health and Environmental Sciences, University of L’Aquila, L’Aquila, Italy

## Abstract

This cross-sectional study evaluates whether urinary iodine concentration is associated with testosterone among men who participated in the National Health and Nutrition Examination Survey.

## Introduction

According to World Health Organization reports, due to iodization policies, iodine intake has long been adequate or even excessive in many countries worldwide, so communities could be at risk of possible adverse effects of excess iodine.^1^ Iodine transporters, namely sodium/iodide symporter and pendrin, have been identified in extrathyroidal tissues, including testis.^2,3^ While both iodine deficiency and excess are associated with poorer seminal quality and longer time to pregnancy,^4^ to our knowledge no information exists on the relationship between iodine exposure and testosterone in humans. This study used a nationally representative sample of the US population from the National Health and Nutrition Examination Survey (NHANES) to explore the association between urinary iodine concentration (UIC) and testosterone levels.

## Methods

In this cross-sectional study, we used 5 cycles of NHANES that included data on total testosterone (TT) and UIC (1999-2002 and 2011-2016), which are publicly available on the US Centers for Disease Control and Prevention website. The NHANES was approved by the National Center for Health Statistics ethics review board, and participants provided written consent. We followed the Strengthening the Reporting of Observational Studies in Epidemiology (STROBE) reporting guideline. UIC and TT levels were determined by inductively coupled plasma dynamic reaction cell mass spectroscopy and isotope dilution high-performance liquid chromatography–tandem mass spectrometry, respectively. Free testosterone was calculated (cFT) with the Vermeulen formula. Overall, 11 433 male participants for whom TT levels were available were surveyed. After excluding 7536 participants due to missing UIC data and 963 participants under the age of 18 years, 2934 men were matched. No differences were found between included and excluded populations (eTable in Supplement 1). Differences among UIC groups were evaluated with Wilcoxon rank-sum test or χ^2^ test, as appropriate. Covariates included in univariate regressions were chosen based on prior pathophysiological knowledge. Multivariable linear regression analysis on log-transformed values assessed the independent association between continuous UIC and TT levels, after adjustment for variables exhibiting univariate associations with TT.

We used R statistical software version 4.2.2 (R Project for Statistical Computing). A 2-tailed *P* ≤ .05 indicated statistical significance.

## Results

A total of 2934 men were included (mean [SD] age, 47.1 [18.4] years). As shown in Table 1, men with low UIC had higher TT and cFT levels than those with normal and high UIC. The low-UIC group was younger and had a more favorable metabolic and glycolipid profile, including lower body mass index. Differences were also found in ethnicity, alcohol intake, education, marital status, and creatinine levels (Table 1).

**Table 1.** Characteristics of the Study Population Stratified by Urinary Iodine Concentration

Characteristic	Participants by UIC level, No. (%)			*P* value		
	Low (<100 μg/L) (n = 997)	Normal (100-299 μg/L) (n = 1437)	High (≥300 μg/L) (n = 500)	Low vs normal UIC	Low vs high UIC	Normal vs high UIC
Age, median (IQR), y	44.10 (31.10-59.10)	47.10 (31.10-64.10)	48.60 (34.10-65.10)	<.001	<.001	.08
Ethnicity^a^						
Mexican American	137 (13.74)	235 (16.35)	68 (13.60)	.08	.09	.14
Non-Hispanic Black	219 (21.97)	322 (22.41)	93 (18.60)	.79	.13	.07
Non-Hispanic White	366 (36.71)	554 (38.55)	235 (47.00)	.35	<.001	<.001
Other ethnicity	275 (27.58)	326 (22.69)	104 (20.80)	.006	.004	.38
Alcohol users^b^	765 (83.37)	1056 (80.79)	342 (76.34)	.07	<.001	.03
Physically active^c^	411 (41.30)	597 (41.60)	193 (38.83)	.87	.33	.24
Education level^d^						
Less than grade 9	94 (9.90)	153 (10.99)	68 (13.85)	.33	.01	.07
Grade 9-11	117 (12.32)	194 (13.94)	59 (12.02)	.19	.97	.33
High school graduate or GED	206 (21.68)	290 (20.83)	111 (22.60)	.77	.61	.43
Some college or advanced degree	278 (29.26)	409 (29.38)	128 (26.07)	.75	.35	.22
College graduate or higher	255 (26.84)	346 (24.86)	125 (25.46)	.39	.81	.68
Marital status^e^						
Married	511 (53.68)	786 (57.25)	265 (54.87)	.09	.52	.51
Divorced, widowed, or separated	114 (11.97)	196 (14.27)	74 (15.32)	.11	.06	.52
Never married	234 (24.58)	271 (19.74)	110 (22.77)	.006	.52	.13
Living with partner	93 (9.77)	120 (8.74)	34 (7.04)	.40	.09	.27
BMI, median (IQR)	27.00 (24.00-30.60)	27.90 (24.60-31.70)	27.50 (24.65-31.25)	<.001	.06	.32
SBP, median (IQR), mm Hg	122.10 (114.10-134.10)	124.10 (114.10-134.10)	124.10 (114.10-136.10)	.42	.34	.64
DBP, median (IQR), mm Hg	72.10 (64.10-80.10)	72.10 (64.10-78.10)	72.10 (64.10-80.10)	.43	.84	.42
TT, median (IQR), ng/dL	446.70 (325.00-539.00)	398.68 (295.00-534.00)	398.50 (288.75-531.25)	.01	.01	.58
SHBG, median (IQR), μg/mL^f^	4.19 (3.04-5.92)	4.02 (2.88-5.92)	4.18 (2.88-6.23)	.55	.97	.63
cFT median (IQR), pg/ml^f^	75.80 (59.25-97.50)	72.10 (53.92-96.50)	67.20 (52.30-94.10)	.04	.004	.17
TSH, median (IQR), mIU/L^g^	1.55 (1.20-2.28)	1.53 (1.12-2.32)	1.69 (1.21-2.38)	.09	.61	.24
Thyroid dysfunction^g^	8 (2.37)	14 (2.28)	12 (5.48)	>.99	.09	.03
Fasting glucose, median (IQR), mg/dL	94.10 (86.10-105.10)	95.10 (87.10-108.10)	94.10 (86.60-107.10)	.005	.31	.31
Triglycerides, median (IQR), mg/dL	125.10 (79.10-185.35)	127.60 (82.10-207.35)	122.10 (81.10-211.60)	.09	.27	.92
Total cholesterol, median (IQR), mg/dL	189.10 (160.10-217.10)	185.60 (159.10-216.35)	179.10 (154.60-207.10)	.49	.001	.004
LDL-c, median (IQR), mg/dL	163.22 (128.00-202.82)	162.91 (129.00-200.47)	154.00 (120.32-196.72)	.80	.04	.01
HDL-c, median (IQR), mg/dL	48.10 (39.10-57.10)	45.10 (38.10-54.10)	45.10 (38.10-54.10)	<.001	.002	.97
Creatinine, median (IQR), mg/dL	1.04 (0.93-1.15)	1.08 (0.95-1.21)	1.08 (0.97-1.22)	<.001	<.001	.63

Abbreviations: BMI, body mass index (calculated as weight in kilograms divided by height in meters squared); cFT, calculated free testosterone; DBP, diastolic blood pressure; GED, General Educational Development Test; HDL-c, high-density lipoprotein cholesterol; LDL-c, low-density lipoprotein cholesterol; SBP, systolic blood pressure; SHBG, sex hormone-binding globulin; TSH, thyroid-stimulating hormone; TT, total testosterone; UIC, urinary iodine concentration.

SI conversion factors: To convert cFT to picomoles per liter, multiply by 3.467; creatinine to micromoles per liter, multiply by 88.4; fasting glucose to millimoles per liter, multiply by 0.555; SHBG to nanomoles per liter, multiply by 8.896; total cholesterol, LDL-c, and HDL-c to millimoles per liter, multiply by 0.0259; triglycerides to millimoles per liter, multiply by 0.0113; TT to nanomoles per liter, multiply by 0.0347.

^a^ Self-reported according to fixed categories. Other ethnicity includes non-Hispanic Asian and multiracial individuals. UIC values [medians (IQR)] among ethnicity groups were as follows: Mexican American, 144.35 (85.70-239.65) μg/L; non-Hispanic Black, 131.20 (83.25-235.37) μg/L; non-Hispanic White, 151.00 (81.45-264.10) μg/L; and other ethnicity, 127.60 (68.70-225.60) μg/L (*P* = .001 with the Kruskal-Wallis test).

^b^ Alcohol users were consumers of at least 12 drinks per year. Data available for 914 men with low UIC, 1307 men with normal UIC, and 448 men with high UIC.

^c^ Physical activity included any moderate-intensity activity in a typical week. Data available for 995 men with low UIC, 1435 men with normal UIC, and 497 men with high UIC.

^d^ Data available for 950 men with low UIC, 1392 men with normal UIC, and 491 men with high UIC. UIC values [medians (IQR)] among education level groups were as follows: less than grade 9, 163.30 (86.75-284.55) μg/L; grade 9-11, 150.25 (83.75-244.27) μg/L; high school graduate or GED, 138.50 (79.35-238.05) μg/L; some college or advanced degree, 137.20 (81.00-242.00) μg/L; college graduate or higher, 135.60 (78.35-243.52) μg/L (*P* = .12 with the Kruskal-Wallis test).

^e^ Data available for 952 men with low UIC, 1373 men with normal UIC, and 483 men with high UIC. UIC values (median [IQR]) among marital status groups were as follows: married, 146.30 (83.70-248.10) μg/L; divorced, widowed, or separated, 151.95 (87.35-264.10) μg/L; never married, 128.10 (69.40-231.60) μg/L; living with partner, 125.10 (65.10-208.10) μg/L (*P* = .001 with the Kruskal-Wallis test).

^f^ Data available for 675 men with low UIC, 980 men with normal UIC, and 333 men with high UIC.

^g^ Thyroid dysfunction included both hyperthyroidisms and hypothyroidisms in their overt (abnormal TSH, free triiodothyronine, and free thyroxine) or subclinical (abnormal TSH and normal both free triiodothyronine and free thyroxine) forms. According to the National Health and Nutrition Examination Survey reference range, normal functional thyroid profile was defined as follows: TSH levels from 0.24 to 5.4 mIU/L, free triiodothyronine from 2.5 to 3.9 pg/mL, and free thyroxine from 0.6 to 1.6 μg/dL. Thyroid data were available for 337 men with low UIC, 613 men with normal UIC, and 219 men with high UIC.

In the multivariable linear regression models including covariates selected by univariate analyses, the negative association between UIC and TT persisted after full adjustment (Table 2). When analysis was repeated by entering cFT as the dependent variable, cFT was negatively associated with UIC in the fully adjusted model (β = −0.031; 95% CI, −0.056 to −0.005).

**Table 2.** Association Between UIC and Total Testosterone Levels: Univariate (Unadjusted) and Multivariable (Adjusted) Linear Regression Analyses^a^

Variable	Unadjusted		Adjusted				
	β coefficient (95% CI), ng/dL^b^	*P* value	Model 1^c^		Model 2^d^		
			β coefficient (95% CI), ng/dL^b^	*P* value	β coefficient (95% CI), ng/dL^b^	*P* value	VIF^e^
UIC (μg/L)	−0.040 (−0.061 to −0.019)	<.001	−0.027 (−0.049 to −0.006)	.01	−0.028 (−0.049 to −0.006)	.01	1.04
Age, y	−0.253 (−0.292 to −0.213)	<.001	−0.209 (−0.262 to −0.155)	<.001	−0.45 (−0.518 to −0.381)	<.001	2.21
Ethnicity^f^							
Mexican American	[Reference]	NA	[Reference]	NA	[Reference]	NA	NA
Non-Hispanic Black	0.023 (−0.036 to 0.082)	.45	0.01 (−0.051 to 0.07)	.75	−0.022 (−0.086 to 0.043)	.51	2.12
Non-Hispanic White	−0.052 (−0.105 to 0.001)	.05	−0.035 (−0.089 to 0.019)	.21	−0.064 (−0.119 to −0.01)	.02	2.27
Other ethnicity	−0.035 (−0.093 to 0.022)	.23	−0.042 (−0.101 to 0.016)	.16	−0.018 (−0.076 to 0.039)	.53	2.06
Marital status							
Married	[Reference]	NA	[Reference]	NA	[Reference]	NA	NA
Divorced, widowed, or separated	0.03 (−0.024 to 0.084)	.27	0.044 (−0.01 to 0.097)	.11	0.053 (−0.002 to 0.107)	.06	1.11
Never married	0.202 (0.157 to 0.247)	<.001	0.091 (0.038 to 0.143)	.001	−0.018 (−0.072 to 0.035)	.50	1.48
Living with partner	0.102 (0.038 to 0.166)	.002	0.018 (−0.048 to 0.085)	.59	−0.046 (−0.11 to 0.018)	.16	1.21
Alcohol intake, yes	0.006 (−0.014 to 0.027)	.52	NA	NA	NA	NA	NA
Physical activity, yes	0.008 (−0.007 to 0.024)	.28	NA	NA	NA	NA	NA
Education level							
Less than grade 9	[Reference]	NA	NA	NA	NA	NA	NA
Grade 9-11	0.045 (−0.027 to 0.118)	.22	NA	NA	NA	NA	NA
High school graduate or GED	0.043 (−0.023 to 0.109)	.19	NA	NA	NA	NA	NA
Some college or advanced degree	−0.003 (−0.066 to 0.059)	.92	NA	NA	NA	NA	NA
College graduate or higher	−0.002 (−0.066 to 0.061)	.94	NA	NA	NA	NA	NA
BMI	−0.877 (−0.958 to −0.797)	<.001	NA	NA	−0.293 (−0.395 to −0.19)	<.001	1.28
SBP	−0.561 (−0.697 to −0.425)	<.001	NA	NA	−0.158 (−0.308 to −0.008)	.039	1.23
DBP	0.038 (0.003 to 0.072)	.03	NA	NA	0.038 (−0.001 to 0.076)	.054	1.02
SHBG	0.327 (0.290 to 0.364)	<.001	NA	NA	0.48 (0.436 to 0.524)	<.001	1.70
Fasting glucose	−0.349 (−0.415 to −0.285)	.001	NA	NA	−0.048 (−0.117 to 0.021)	.17	1.18
Triglycerides	−0.206 (−0.232 to −0.18)	<.001	NA	NA	−0.04 (−0.074 to −0.006)	.02	1.74
HDL-c	0.359 (0.297 to 0.421)	<.001	NA	NA	0.038 (−0.039 to 0.115)	.34	1.66
Creatinine	−0.128 (−0.212 to −0.444)	.003	NA	NA	−0.044 (−0.132 to 0.043)	.32	1.18
Thyroid dysfunction, yes	−0.024 (−0.100 to 0.052)	.53	NA	NA	NA	NA	NA

Abbreviations: BMI, body mass index; DBP, Diastolic blood pressure; HDL-c, high-density lipoprotein cholesterol; NA, not applicable; SHBG, sex hormone binding globulin; SBP, Systolic blood pressure; UIC, urinary iodine concentration; VIF, variance inflation factors.

^a^ Analyses used the original continuous scale of total testosterone levels in nanograms per deciliter and UIC in microgram per liter.

^b^ To interpret the β coefficient of log-transformed data, a 1% increase in independent variable is associated with an average change of β per 100 units in dependent variable (total testosterone in nanograms per deciliter).

^c^ Model 1 was adjusted for demographic variables selected by univariate analysis (age, ethnicity, and marital status).

^d^ Model 2 was adjusted for model 1 with metabolic variables selected by univariate analysis (BMI, SBP, DBP, SHBG, fasting glucose, triglycerides, HDL-c, and creatinine).

^e^ Multicollinearity in the fully adjusted model was ruled out for VIF less than 5.

^f^ Self-reported according to fixed categories. Other ethnicity includes non-Hispanic Asian and multiracial individuals.

## Discussion

In this study, lower UIC was associated with higher testosterone levels independent of factors that may influence androgen status, including age and features of metabolic syndrome, whose prevalence is positively associated with UIC.^5^ This unprecedented evidence deserves to be clarified in its causal directionality. While an interaction between iodine intake, thyroid hormones, and testosterone cannot be definitively ruled out, excess iodine could affect testicular steroidogenesis directly. In studies with rats, after a high iodine–diet feeding, iodine accumulates in the testis and triggers oxidative stress that inhibits 3β- and 17β-hydroxysteroid dehydrogenase activity, thus lowering testosterone synthesis.^6^

Study limitations include the cross-sectional design that does not establish cause-effect relationships, residual unmeasured confounding, and unavailability of gonadotropin measurements that could have pointed toward the interference level(s). Additionally, UIC may not entirely reflect body iodine status, being influenced by hydration and recent iodine intake.

In conclusion, lower UIC is independently associated with higher testosterone levels. Caution should be paid to excessive iodine supplementation.

## Notes

Supplement 1. **eTable.** Characteristics of the Included Study Population Compared With Adult Men Excluded Due to Missing Urinary Iodine Concentration

Supplement 2.
Data Sharing Statement
