# Supplementary material for: Iodine Intake and Testosterone
Source: JAMA Netw Open. 2023 Dec 20;6(12):e2348573. doi: 10.1001/jamanetworkopen.2023.48573 (PMC10733805; doi:10.1001/jamanetworkopen.2023.48573)
Supplement: Supplement 1. — eTable. Characteristics of the Included Study Population Compared With Adult Men Excluded Due to Missing Urinary Iodine Concentration [file jamanetwopen-e2348573-s001.pdf]

Supplemental Online Content

Barbonetti A, Castellini C, Di Giulio F, et al. Iodine intake and testosterone. *JAMA Netw Open*. 2023;6(12):e2348573. doi:10.1001/jamanetworkopen.2023.48573

eTable. Characteristics of the Included Study Population Compared With Adult Men Excluded Due to Missing Urinary Iodine Concentration

This supplemental material has been provided by the authors to give readers additional information about their work.

© 2023 Barbonetti A et al. *JAMA Network Open.*

eTable. Characteristics of the Included Study Population Compared With Adult Men Excluded Due to Missing Urinary Iodine Concentration

Characteristics, No. (%)	Included (N = 2934)	Excluded (N = 5462)[a]	P value
Age, years	46.00 (31.00)	47.10 (32.00)	.14
Ethnicity[b]			
Mexican American	440 (15.00)	827 (15.14)	.88
Non-Hispanic Black	634 (21.61)	1153 (21.11)	.61
Non-Hispanic White	1155 (39.36)	2126 (38.92)	.71
Other Ethnicity[b]	705 (24.03)	1356 (24.83)	.43
Alcohol[c]	2163 (81.04)	4078 (81.00)	.98
Physically active[d]	1201 (41.03)	2318 (42.44)	.22
Education level[e]			
Less than grade 9	315 (11.12)	542 (10.25)	.24
Grade 9-11	370 (13.06)	711 (13.45)	.65
High school graduate or GED	607 (21.42)	1177 (22.26)	.40
Some college or advanced degree	815 (28.77)	1511 (28.58)	.88
College graduate or higher	726 (25.63)	1346 (25.46)	.89
Marital status[f]			
Married	1562 (55.63)	2900 (56.00)	.77
Divorced, widowed, or separated	384 (13.67)	791 (15.27)	.06
Never married	615 (21.90)	1051 (20.29)	.10
Living with partner	247 (8.80)	437 (8.44)	.61
BMI, median (IQR), Kg/m^2	27.50 (6.82)	27.66 (7.30)	.61
SBP, median (IQR), mmHg	124.10 (20.00)	124.00 (20.00)	.32
DBP, median (IQR), mmHg	72.00 (16.00)	72.00 (14.00)	.20
TT, median (IQR), ng/dL	405.18 (229.34)	404.00 (230.81)	.44
TSH, median (IQR), mIU/L[g]	1.56 (1.18)	1.69 (0.98)	.63
Thyroid dysfunction[g]	34 (2.91)	9 (3.41)	.82
Fasting glucose, median (IQR), mg/dL	94.00 (20.00)	95.10 (20.00)	.22
Triglycerides, median (IQR), mg/dL	126.00 (120.00)	125.00 (119.00)	.53
HDL-c, median (IQR), mg/dL	46.10 (16.00)	46.00 (17.00)	.42
Creatinine, median (IQR), mg/dL	1.06 (0.24)	1.05 (0.25)	.71

Abbreviations: BMI, body mass index; DBP, Diastolic blood pressure; GED, General Educational Development Test; HDL-c, high-density lipoprotein cholesterol; IQR, interquartile range; SBP, systolic blood pressure; TSH, thyroid-stimulating hormone; TT, total testosterone.
Système International (SI) conversion factors: TT: from ng/dL to nmol/L, multiply by 0.035; fasting glucose: from mg/dL to mmol/L, multiply by 0.555; triglycerides: from mg/dL to mmol/L multiply by 0.0113; HDL-c: from mg/dL to mmol/L multiply by 0.0259; creatinine: from mg/dL to µmol/L multiply by 88.4.
[a]From the initial cohort of 7536 male participants lacking of urinary iodine concentration measurement, 2074 participants under the age of 18 were excluded.
[b]Self-reported according to fixed categories. Other Ethnicity includes non-Hispanic Asian and multiracial individuals.
[c]data available in 2669 included men and 5035 excluded men.
[d]data available in 2927 included men and 5462 excluded men.
[e]data available in 2833 included men and 5287 excluded men.
[f]data available in 2808 included men and 5179 excluded men.
[g]Thyroid dysfunction included both hyperthyroidisms and hypothyroidisms in their overt (abnormal TSH, free triiodothyronine and free thyroxine) or subclinical (abnormal TSH and normal both free triiodothyronine and free thyroxine) forms. According to NHANES reference ranges, normal functional thyroid profile was defined as follows: TSH levels from 0.24 to 5.4 mIU/L, free triiodothyronine from 2.5 to 3.9 pg/mL, and free thyroxine from 0.6 to 1.6 µg/dL. Thyroid data were available in 1169 included men and 264 excluded men.

© 2023 Barbonetti A et al. *JAMA Network Open.*
