# Supplementary material for: Iodine Intake and Testosterone
Source: JAMA Netw Open. 2023 Dec 20;6(12):e2348573. doi: 10.1001/jamanetworkopen.2023.48573 (PMC10733805; doi:10.1001/jamanetworkopen.2023.48573)
Supplement: Supplement 2. — Data Sharing Statement [file jamanetwopen-e2348573-s002.pdf]

Data Sharing Statement

Barbonetti. Iodine Intake and Testosterone. *JAMA Netw Open*. Published December 20, 2023.
doi:10.1001/jamanetworkopen.2023.48573

Data
Data available: Yes
Data types: Deidentified participant data
How to access data: NHANES data are deidentified and publicly available. All data are publicly available at https://www.cdc.gov/nchs/nhanes/index.htm
When available: With publication

Supporting Documents
Document types: None

Additional Information
Who can access the data: All data are publicly available to all researchers
Types of analyses: For any purpose
Mechanisms of data availability: All data are publicly available to all researchers
Any additional restrictions: N/A
